# Flexible Stoichiometry and Asymmetry of the PIDDosome Core Complex by Heteronuclear NMR Spectroscopy and Mass Spectrometry

**DOI:** 10.1016/j.jmb.2014.11.021

**Published:** 2015-02-27

**Authors:** Lily A. Nematollahi, Acely Garza-Garcia, Chérine Bechara, Diego Esposito, Nina Morgner, Carol V. Robinson, Paul C. Driscoll

**Affiliations:** 1 Division of Molecular Structure, Medical Research Council, National Institute for Medical Research, The Ridgeway, Mill Hill, London NW7 1AA, UK; 2 Department of Chemistry, Physical and Theoretical Chemistry Laboratory, University of Oxford, Oxford OX1 3QZ, UK

**Keywords:** CARD, caspase recruitment domain, DD, death domain, nanoESI, nanoflow electrospray ionization, MS, mass spectrometry, HSQC, heteronuclear single quantum coherence, SEC, size-exclusion chromatography, MALS, multi-angle light scattering, TCEP, tris(2-carboxyethyl)phosphine, TROSY, transverse relaxation optimized spectroscopy, 2D, two-dimensional, death domain, ILV-labeling, protein complex, TROSY NMR, spectral complexity

## Abstract

Homotypic death domain (DD)–DD interactions are important in the assembly of oligomeric signaling complexes such as the PIDDosome that acts as a platform for activation of caspase-2-dependent apoptotic signaling. The structure of the PIDDosome core complex exhibits an asymmetric three-layered arrangement containing five PIDD-DDs in one layer, five RAIDD-DDs in a second layer and an additional two RAIDD-DDs. We addressed complex formation between PIDD-DD and RAIDD-DD in solution using heteronuclear nuclear magnetic resonance (NMR) spectroscopy, nanoflow electrospray ionization mass spectrometry and size-exclusion chromatography with multi-angle light scattering. The DDs assemble into complexes displaying molecular masses in the range 130–158 kDa and RAIDD-DD:PIDD-DD stoichiometries of 5:5, 6:5 and 7:5. These data suggest that the crystal structure is representative of only the heaviest species in solution and that two RAIDD-DDs are loosely attached to the 5:5 core. Two-dimensional ^1^H,^15^N-NMR experiments exhibited signal loss upon complexation consistent with the formation of high-molecular-weight species. ^13^C-Methyl-transverse relaxation optimized spectroscopy measurements of the PIDDosome core exhibit signs of differential line broadening, cross-peak splitting and chemical shift heterogeneity that reflect the presence of non-equivalent sites at interfaces within an asymmetric complex. Experiments using a mutant RAIDD-DD that forms a monodisperse 5:5 complex with PIDD-DD show that the spectroscopic signature derives from the quasi- but non-exact equivalent environments of each DD. Since this characteristic was previously demonstrated for the complex between the DDs of CD95 and FADD, the NMR data for this system are consistent with the formation of a structure homologous to the PIDDosome core.

## Highlights

• The PIDDosome core particle that has been crystallized as a 7:5 complex displays heterogeneous stoichiometry in solution.
• Methyl-transverse relaxation optimized spectroscopy NMR spectra for the complex suggest that individual PIDD-DDs and RAIDD-DDs experience non-equivalent environments in the PIDDosome core.
• A mutant PIDDosome core particle that is monodisperse displays similar NMR features, suggesting that the complexity of the spectra is a reflection of the absence of formal symmetry consistent with the crystal structure.
• The NMR characteristics are reminiscent of those reported for the complex formed between the DDs of CD95 and FADD, suggesting that this latter complex has similar architecture to the PIDDosome core.

## Graphical Abstract

**Figure:**
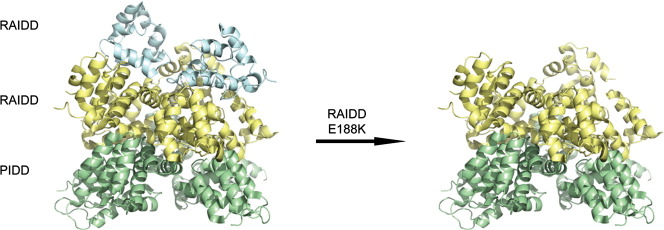

**Legend:**
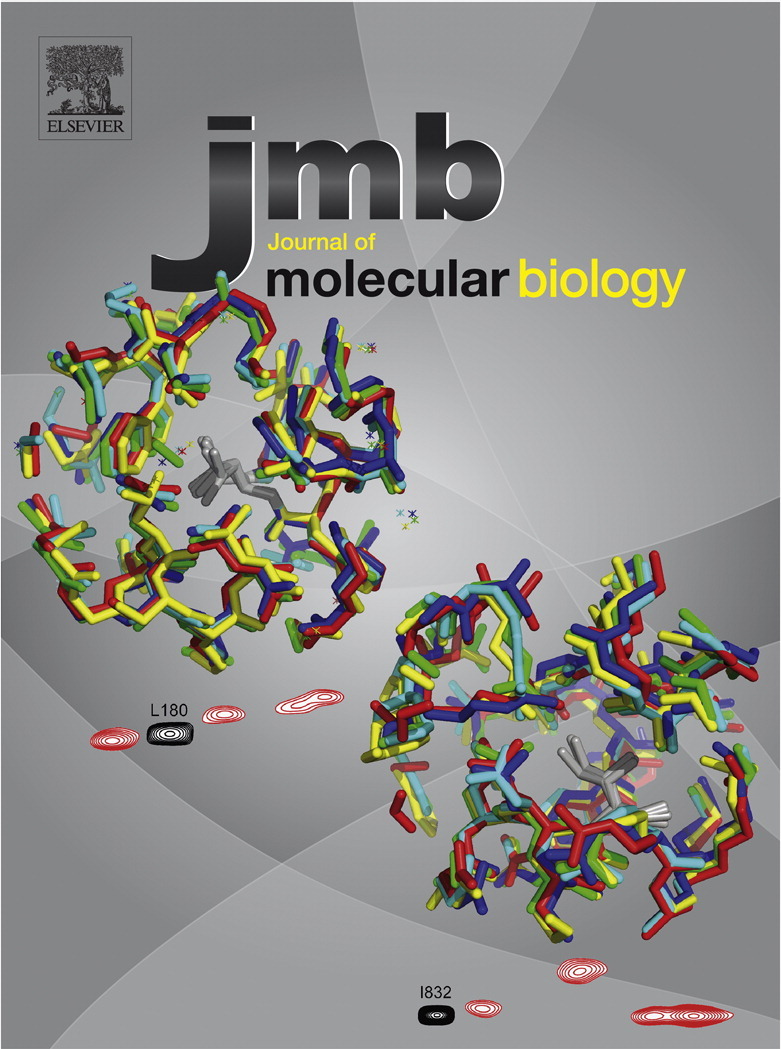
Non-equivalence of methyl-group side chains (gray) among the pseudoequivalent interdomain contacts in the PIDDosome core crystal structure (PDB code 2OF5) is reflected in the distribution of the transverse relaxation optimized spectroscopy nuclear magnetic resonance intensity observed as a single feature for the isolated PIDD death domain (DD) (black contours) into multiple features in the spectrum of the complex (red contours).

## Introduction

**Fig. 1:**
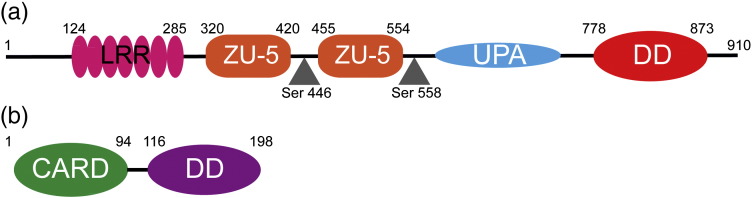
Schematic representation of the domain structure (a) PIDD and (b) RAIDD proteins, with predicted domain boundaries indicated in numerals. For PIDD, the gray triangles indicate the points at which intein-like proteolytic autocleavage leads to C-terminal fragments of the protein.

The PIDDosome is a high-molecular-weight cytosolic complex that is formed as a response to DNA damage or cellular stress containing three proteins: p53-induced DD protein (PIDD), receptor-interacting protein-associated Ich-1/CED homologous protein with DD (RAIDD) and the cysteine protease caspase-2 [1–3]. The PIDDosome acts as a platform for activation of caspase-2 and triggering of the mitochondrial apoptotic pathway [4–6]. PIDD contains seven leucine-rich repeats at the N-terminus; two ZO-1 and Unc5-like (ZU-5) domains; an uncharacterized protein domain in Unc5, PIDD and ankyrin family of proteins (UPA) domain; and a DD (PIDD-DD) at the C-terminus (Fig. 1a) [7,8]. PIDD undergoes autocleavage in an intein-like mode to yield fragments of differing molecular masses: cleavage at Ser446 in the linker between the ZU-5 domains yields PIDD-N (residues 1–445, 48 kDa) and PIDD-C (residues 446–910, 51 kDa); further cleavage of PIDD-C at Ser588 in the linker between the second ZU-5 domain and the DD yields PIDD-CC (residues 588–910, 37 kDa) [7,9,10]. PIDD-C can form a complex with receptor-interacting protein 1 and NF-κB essential modulator, leading to the activation of the transcription factor NF-κB. The PIDD-CC fragment is required for the RAIDD-dependent activation of caspase-2. RAIDD therefore acts as an adaptor protein between PIDD-CC and caspase-2, comprising an N-terminal caspase recruitment domain (CARD) tethered to a C-terminal DD (RAIDD-DD) (Fig. 1b) [2]. Caspase-2, the enzymatic component of the PIDDosome complex, possesses an extended N-terminal CARD domain and a C-terminal protease domain composed of large (p19) and small (p12) subunits [11,12]. It is reported that the expression of just the PIDD-DD is sufficient to sensitize colon carcinoma cells to UV-induced apoptosis [10] and to induce formation of a complex with RAIDD and caspase-2 in 293T cells [9]. Homotypic interaction between the DDs of PIDD and RAIDD and CARDs of RAIDD and caspase-2 leads to the formation of the PIDDosome that brings caspase-2 molecules into proximity, thereby facilitating dimerization and caspase activation [13]. Caspase-2 permeabilizes the outer mitochondrial membrane resulting in the release of cytochrome *c* and Smac/DIABLO and subsequent activation of executioner caspase-3 and caspase-9 [14]. That PIDD is implicated in both pro-survival (NF-κB) and cell death mechanisms means that it has been labeled as a “switch hitter” [7,15,16] with a potentially complex mode of regulation. For example, it has been suggested that interaction of PIDD with RAIDD and subsequent caspase-2 activation is facilitated by phosphorylation of Thr788, a residue adjacent to the DD [1]. One possibility is that phosphorylation of PIDD Thr788 relieves intramolecular inhibition of the RAIDD-binding potential of PIDD-DD in the full-length protein.

**Fig. 2:**
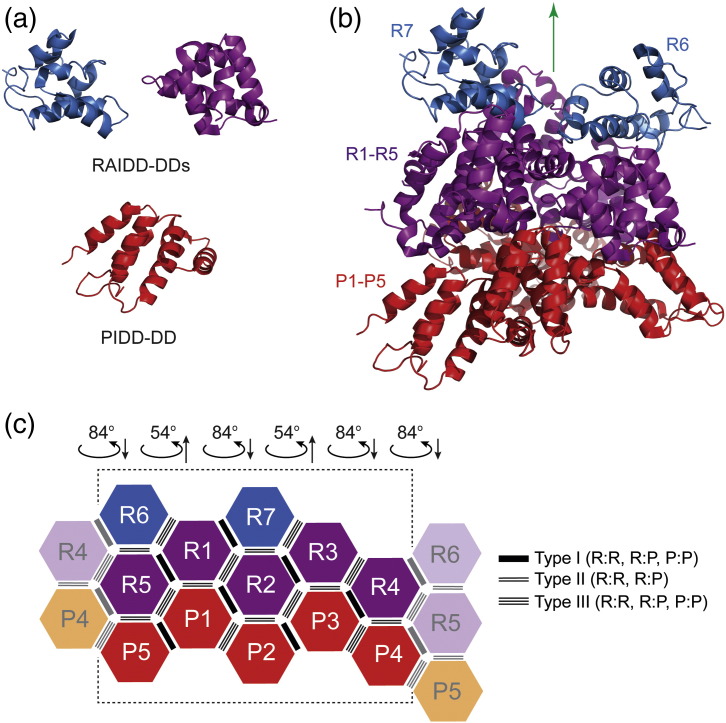
The crystal structure of the PIDDosome complex formed between PIDD-DD and RAIDD-DD (PDB ID 2OF5). Magenta and blue colors are used to depict the RAIDD-DD domains; red, the PIDD-DD domains. (a) Backbone secondary structure of isolated RAIDD-DD and PIDD-DD chains. (b) Overall PIDDosome structure arranged to highlight the bottom layer of five PIDD-DDs, the “middle” layer of five RAIDD-DDs and the top two RAIDD-DDs R6 and R7. (c) A schematic summary of the domain–domain interactions present within the PIDDosome core structure, adapted from Ref. [2]. The broken lines indicate the extent of the core structure, with domains P4, R4 (left-hand side) and P5, R5 and R6 (right-hand side) depicted a second time in lighter hues to represent the axis of pseudohelical symmetry, suggested by the green arrow in (b). Each domain occupies a pseudosimilar position in the structure, defined by the types I–III interdomain contacts [19] respectively indicated by a thick black bar, two parallel lines and three parallel lines. The angular rotation and translational rise and fall reflecting the domain-to-domain packing of the structure are depicted by arrows at the top of the schematic.

*In vitro* reconstitution of the core of the PIDDosome complex has been demonstrated using the isolated DDs of PIDD and RAIDD [2,3,17]. The 3.2-Å-resolution crystal structure (PDB code 2OF5) was obtained for a complex formed between the two DDs at pH 6.5 [2]. The structure revealed a high-molecular-weight particle containing five PIDD-DDs and seven RAIDD-DDs arranged in three layers (Fig. 2). Five PIDD-DD chains occupy a bottom layer that sits underneath a second layer of five RAIDD-DDs; two additional RAIDD-DDs sit on top of these five RAIDD-DDs in a third layer (Fig. 2b). The overall structure is formally asymmetric, lacking any mirror planes. In the structure, each DD has between three and six immediate neighbors. The eight different examples of interdomain interface have been categorized into three classes, known as types I, II and III based on the regions of the DD structure that are involved in each contact [2,18–20]. The whole arrangement of the DDs within the structure can be represented schematically as shown in Fig. 2c.

The crystal structure of the PIDDosome core appears to accurately represent the species present in solution. Prediction of the electron microscopy projections from the structure provides images that closely match those observed experimentally [2]. Moreover, the 150-kDa molecular mass of the particle predicted from the crystal structure is in line with estimates based on solution measurements using size-exclusion chromatography (SEC) coupled with multi-angle light scattering (MALS) [2]. Curiously, however, the molecular mass was separately reported as 103 kDa when assessed by sedimentation equilibrium analytical ultracentrifugation [3,17]. Although the PIDDosome core crystal structure has provided useful information about the interactions and interfaces involved in the formation of the complex, the unusual 7:5 stoichiometry, the absence of formal symmetry and the inconsistent reports of the molecular mass reflect that questions remain concerning the properties of the particle in solution.

Transverse relaxation optimized spectroscopy (TROSY) methods represent a means to probe the nature of high-molecular-weight species in solution. In particular, ^13^C-methyl-group TROSY experiments enable the characterization of the environments of methyl groups in arbitrarily large macromolecular assemblies, for example, see Refs. [21–28]. These experiments exploit the effects of cross-correlation of dipolar ^1^H-^1^H and ^1^H-^13^C interactions within isolated ^1^H_3_^13^C-methyl groups that lead to a component of the magnetization that relaxes slowly and hence leads to relatively sharp cross-peaks. The application of ^13^C-methyl-TROSY requires that the methyl ^13^C atom is bonded to a ^12^C atom and that vicinal protons, in particular, are replaced with deuterons, so as to eliminate deleterious scalar and dipolar coupling interactions. Typically, the required isotope-labeling pattern is obtained by culturing the expression strain of bacteria in a D_2_O/glucose-based minimal medium that contains a perdeuterated carbon source and appropriately labeled amino acid precursor molecules. Incorporation of methyl-^13^C-3,3-*d*_2_-ketobutyric acid leads to ^1^H_3_,^13^C^δ1^-methyl-*d*_10_-isoleucine whereas 3-methyl-^13^C-3,4,4,4-*d*_4_-ketoisovaleric acid leads to both ^1^H_3_,^13^C^γ^-methyl-*d*_8_-valine and ^1^H_3_,^13^C^δ^-methyl-*d*_10_-leucine, wherein only one of the two prochiral methyl groups is ^1^H_3_,^13^C^γ^-labeled. For brevity, protein samples prepared using a combination of these precursors are referred to as ILV-labeled.

Although the ILV-labeling technique has been widely used to study isotope-labeled proteins within symmetric homomeric or heteromeric particles [25,29–36], examples of its application to asymmetric multimers have been extremely limited. We have previously applied ^13^C-methyl-TROSY nuclear magnetic resonance (NMR) to particles assembled by the DDs of the tumor necrosis superfamily receptor CD95/Fas and its adaptor protein FADD [33]. The spectra of the complexes obtained contained many more cross-peaks than were present for the isolated components, leading us to speculate that the particles either were highly heterogeneous in character or might lack formal symmetry, or possibly both.

Here we revisit the structure and composition of the PIDDosome core complex in solution. We establish that the crystal structure is representative of the heaviest PIDDosome core complex species present in solution. A mutation designed to selectively disrupt the type II RAIDD-DD:RAIDD-DD interface leads to the formation of a more uniformly disperse 5:5 subcomplex. We have also applied ILV labeling and ^13^C-methyl-TROSY to the PIDDosome core particle. Remarkably, after controlling for the potential for compositional heterogeneity, we find that the NMR spectra clearly reflect the asymmetry anticipated from the crystal structure, arguing that the complexity of the quaternary structure is not a result of crystal lattice forces. Moreover, the data indicate that exchange between subsites in the PIDDosome core particle is slow on the chemical shift timescale. The observations bolster the interpretation of the previously recorded ^13^C-methyl-TROSY spectra for the CD95/FADD DD assembly in terms of an asymmetric structure rather than compositional heterogeneity [33], as well as reinforce the conclusion that the structure of that particle resembles that of the PIDDosome core [37].

## Results

### The PIDDosome core complex shows flexible stoichiometry in solution and gas phases

**Fig. 3:**
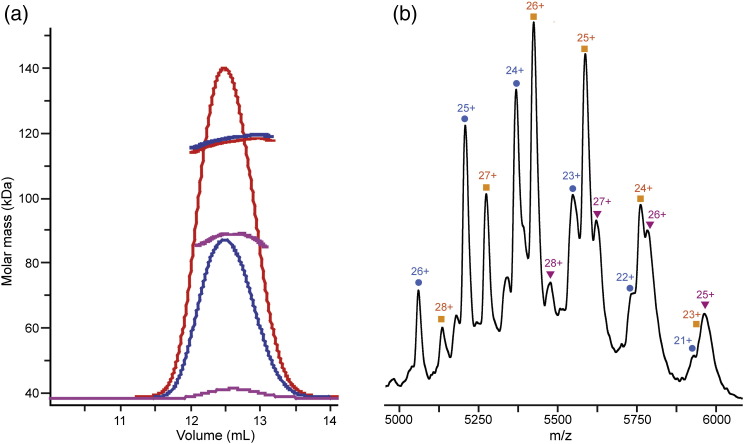
Assessment of the molecular mass of the wild-type RAIDD-DD:PIDD-DD complexes in solution by (a) SEC-MALS and (b) nanoESI-MS. In (a), the overall protein loading concentration was 1.5 mg/mL (magenta), 12 mg/mL (blue) and 24 mg/mL (red). In (b), native mass spectrum of 15 μM complex in 50 mM ammonium acetate buffer (pH 7.8) and ion series corresponding to RAIDD-DD:PIDD-DD species with stoichiometries 5:5, 6:5 and 7:5 are labeled with their charge states and indicated with blue circles, orange squares and purple triangles, respectively.

There are conflicting reports concerning the molecular mass and stoichiometry of the PIDDosome particle [2,3,17]. We analyzed the molecular mass of the complex formed by RAIDD-DD with PIDD-DD using both analytical SEC coupled to MALS and nanoflow electrospray ionization (nanoESI) mass spectrometry (MS) (Fig. 3a).

SEC-MALS samples were run over a total protein concentration range of 1.5–24 mg/mL. The symmetric chromatographic light-scattering profile was consistent with a monodisperse particle with elution volume 12.4 mL in the range 6–24 mg/mL. The elution volume was slightly larger (12.6 mL) at 1.5 mg/mL, suggesting some subunit dissociation at the lowest concentration tested. There was no significant readout at elution volumes greater than 13 mL other than that of a small amount of excess free DD at 17.5 mL (data not shown), indicating the absence of any population corresponding to intermediate species and suggesting a considerable degree of cooperativity in complex formation.

Analysis of the fractions of analytical gel-filtration experiments by SDS-PAGE revealed that the eluted complexes contained approximately equal amounts of the two component DDs (data not shown). The SEC-MALS data indicate that the two DDs form a complex with a maximum molecular mass of ~ 120 kDa. Taking into consideration the predicted molecular masses of the individual domains (PIDD-DD, 13.4 kDa; RAIDD-DD, 13.0 kDa), these results suggest the formation of a particle containing at least nine protein chains. Decreasing the total protein concentration resulted in a reduction in the average molecular mass to 93 kDa (corresponding to a total of seven DD chains).

Purified PIDDosome core complexes were analyzed by nanoESI-MS using procedures designed to maintain non-covalent interactions in complexes [38]. The measurements provide a snapshot of the profile of molecular masses and subunit composition of macromolecular assemblies present in the sample. The mass spectrum obtained shows signals for high *m*/*z* ion series attributable to the PIDDosome core particles. There were three main overlapping charge series of ions. These series represent complexes with an overall mass of 132.0 kDa, 145.1 kDa and 158.1 kDa corresponding to 5:5, 6:5 and 7:5 stoichiometry, respectively, based on internal mass reference for the isolated RAIDD-DD and PIDD-DD monomers (Fig. 3b). The population distributions of the three complexes were 28%, 43% and 29%, respectively, and the recovered molecular masses were within 3% of the predicted values based on the amino acid sequence. There were also some minor peaks in the spectrum corresponding to higher-molecular-weight species that might be due to non-specific binding of higher order. Importantly, ion series corresponding to assemblies with molecular mass lower than 132 kDa were not observed.

### The PIDDosome core complex is mainly invisible to ^15^N-HSQC experiments as expected for a high-molecular-weight particle

**Fig. 4:**
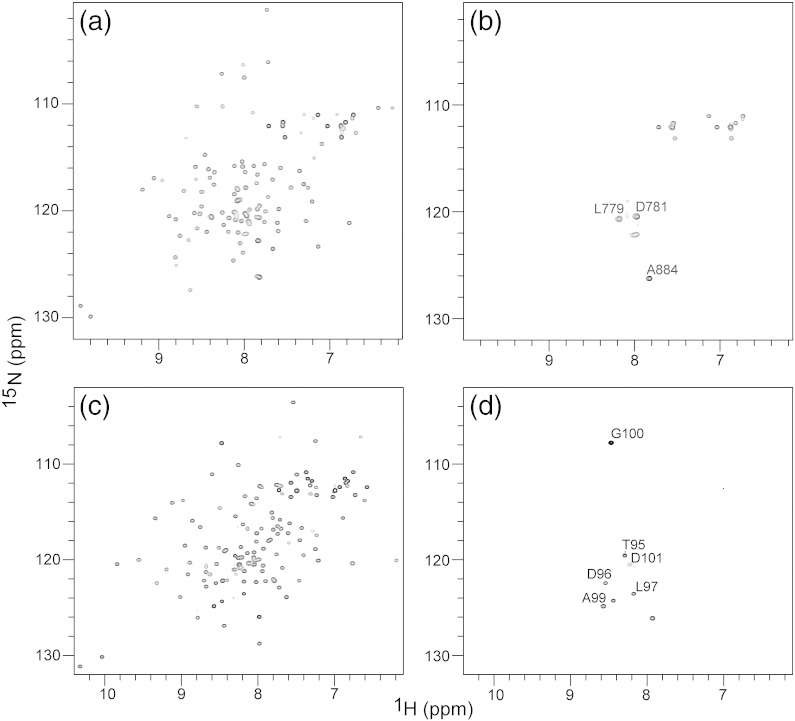
NMR evidence for high-molecular-weight PIDDosome complex formation. Two-dimensional ^15^N-heteronuclear NMR spectra of isolated ^15^N-labeled RAIDD-DD (a) and ^15^N-PIDD-DD (c) and the corresponding complexes formed with excess unlabeled PIDD-DD (b) and RAIDD-DD (d), respectively. For the complexes, only a small number of cross-peaks are detected, and resonance assignment of these signals indicates that they originate from the extreme N- and C-termini of PIDD-DD and RAIDD-DD in each case.

We separately titrated ^15^N-labeled PIDD-DD, as well as ^15^N-labeled RAIDD-DD with the respective unlabeled binding partner, and recorded series of two-dimensional (2D) ^15^N,^1^H-heteronuclear single quantum coherence (HSQC) NMR spectra using the method reported previously [33] (Fig. 4). In all cases, as the concentration of the unlabeled binding partner is increased, the majority of the cross-peaks in the spectrum showed significant reduction in their intensity, ultimately to a level below the noise. Throughout the titration, there is no evidence of chemical shift perturbation of the cross-peaks. Only a small subset of cross-peaks remains at the end of the titration. Sequence-specific resonance assignments were used to confirm that the persistent cross-peaks belong to the protein backbone amide NH groups or side-chain NH_2_ groups of Asn, Gln or Arg residues from the extreme N- and C-terminal regions of each domain type. Thus, the backbone amide cross-peaks of residues Asp781 and Ala884 at the N- and C-terminus, respectively, of PIDD-DD (Fig. 4b) and the backbone amide cross-peaks of Thr95–Asp101 in the N-terminus and a backbone amide NH from the hexahistidine affinity tag in the C-terminus of RAIDD-DD (Fig. 4d) remain visible at the end of the titration.

### ILV-labeling and TROSY-based NMR experiments show complex formation between PIDD-DD and RAIDD-DD

**Fig. 5:**
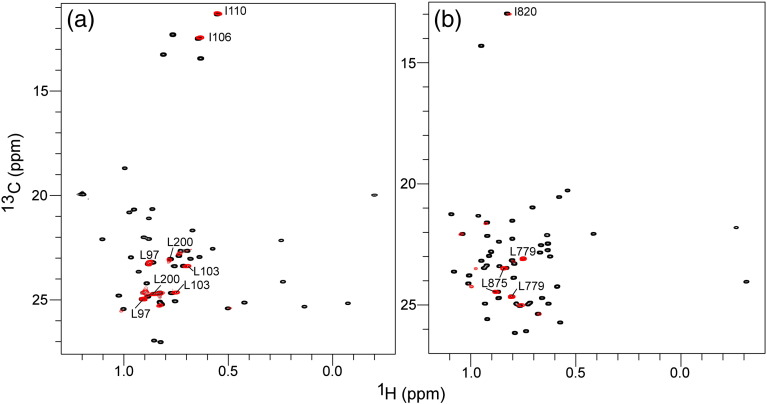
Methyl-TROSY NMR spectra for PIDDosome formation. Two-dimensional ^13^C-methyl-TROSY NMR spectra of ILV-^13^CH_3_-labeled RAIDD-DD (a) and PIDD-DD in the presence (red) and absence (black) of excess unlabeled PIDD-DD and RAIDD-DD, respectively, plotted at high contour threshold. A subset of the assignments of cross-peaks that remain visible in the complexed state is indicated in each case.

We analyzed complex formation between PIDD-DD and RAIDD-DD using ^1^H,^13^C-methyl-group labeling for Ile, Leu and Val side chains in perdeuterated ILV-labeled samples [34] and ^13^C-methyl-TROSY NMR [39]. We separately prepared ILV-^13^CH_3_-labeled perdeuterated samples of RAIDD-DD and PIDD-DD. The corresponding ^13^C-methyl-TROSY spectra are presented in Fig. 5. In each case, the cross-peaks in the free-state spectra are well resolved and display chemical shift dispersion typical for a small globular protein. Addition of a perdeuterated sample of the respective DD binding partner dramatically changed the appearance of the spectrum. When the unlabeled protein is added to ILV-^13^CH_3_-labeled binding partner, the intensity of the methyl-group cross-peaks is significantly reduced due to the complex formation. However, the effects on the cross-peaks can be divided into two characteristically distinguishable types. First, a small subset of cross-peaks retains their narrow line shape, with relatively high peak intensity, and either unperturbed or only slightly perturbed chemical shifts relative to those for the unbound protein. This subset of cross-peaks, which are only marginally broader in the spectrum of the complex compared to the free-state spectrum, can readily be identified by plotting the spectrum at a relatively high base contour level. Specific resonance assignment of these cross-peaks showed that they can be attributed to the ILV residues residing in the flexible N- and C-terminal regions of the labeled protein. For example, the methyl-group cross-peaks corresponding to Ile106, Ile110, Leu103 and Leu97 in ILV-labeled RAIDD-DD are members of this subset and correspond to residues in the flexible N-terminus. Similarly, a methyl-group cross-peak for Leu200 that resides in the vector-encoded C-terminal affinity tag in RAIDD-DD is also in this group (Fig. 5a). Correspondingly, the methyl-group cross-peaks for Leu779 and Leu875, respectively arising from the flexible N- and C-termini of PIDD-DD, show essentially identical characteristics in the presence of perdeuterated RAIDD-DD (Fig. 5b).

**Fig. 6:**
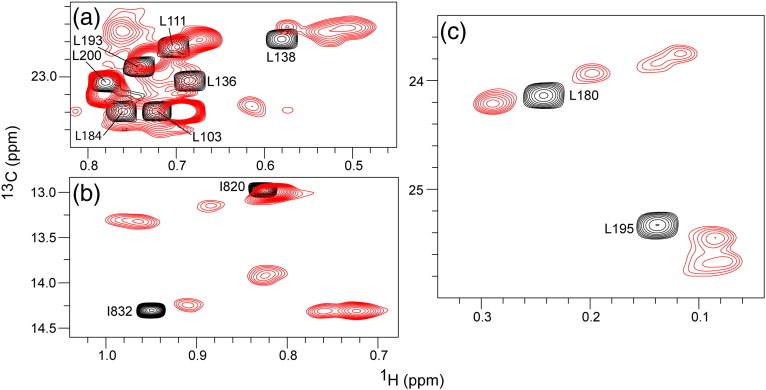
Cross-peak clusters are formed for many methyl groups upon PIDDosome formation. Selected regions of the ^13^C-methyl-TROSY NMR spectra of ILV-^13^CH_3_-labeled RAIDD-DD (a and c) and PIDD-DD (b) in the presence (red) and absence (black) of excess unlabeled PIDD-DD and RAIDD-DD, respectively, plotted at low contour threshold to reveal the clusters of low-intensity cross-peaks associated with PIDDosome formation. Methyl-group assignments of the cross-peaks in the unbound state are indicated.

The remaining ILV-methyl-group cross-peaks are only visible at much lower contour levels. As exemplified in Fig. 6, these cross-peaks show a distinctly different dependence upon the presence of the binding partner protein. Namely, it appears that each cross-peak in the free-state spectrum splits into a cluster of either resolved or partially overlapping cross-peaks in the spectrum of the complex. The total intensity of each starting “singlet” cross-peak is apparently dispersed among the multiple members of the resultant clusters. The complexity of the spectrum is dramatically increased as a result of the increased cross-peak multiplicity particularly in the middle of the spectrum (Fig. 6a), making identification of the link between parent “singlet” and daughter cluster components ambiguous. The precise nature of the effect of complex formation on the ^13^C-methyl-TROSY spectrum is most readily understood by examining the fates of the most highly resolved features in the free-state spectrum. For instance, the “singlet” cross-peaks for Ile820-C^δ1^H_3_ and Ile832-C^δ1^H_3_ in ILV-labeled PIDD-DD (Fig. 6b) and Val180-C^γ1^H_3_ and Val195-C^γ2^H_3_ in ILV-labeled RAIDD-DD (Fig. 6c) are each converted into a cluster of at least four separate cross-peaks, with differing intensity, in the spectrum of the corresponding complex.

The splitting of the methyl-group cross-peaks into clusters in the presence of the binding partner must result from the formation of one or more structures in which the environment of individual component molecules is non-equivalent. Such an outcome could arise from the formation of a single asymmetric structure or potentially from the presence of multiple complexes with different composition. In principle, both scenarios might apply together; that is, sample contains different complexes each with a different symmetry.

### Design of a monodisperse 5:5 PIDDosome complex

**Fig. 7:**
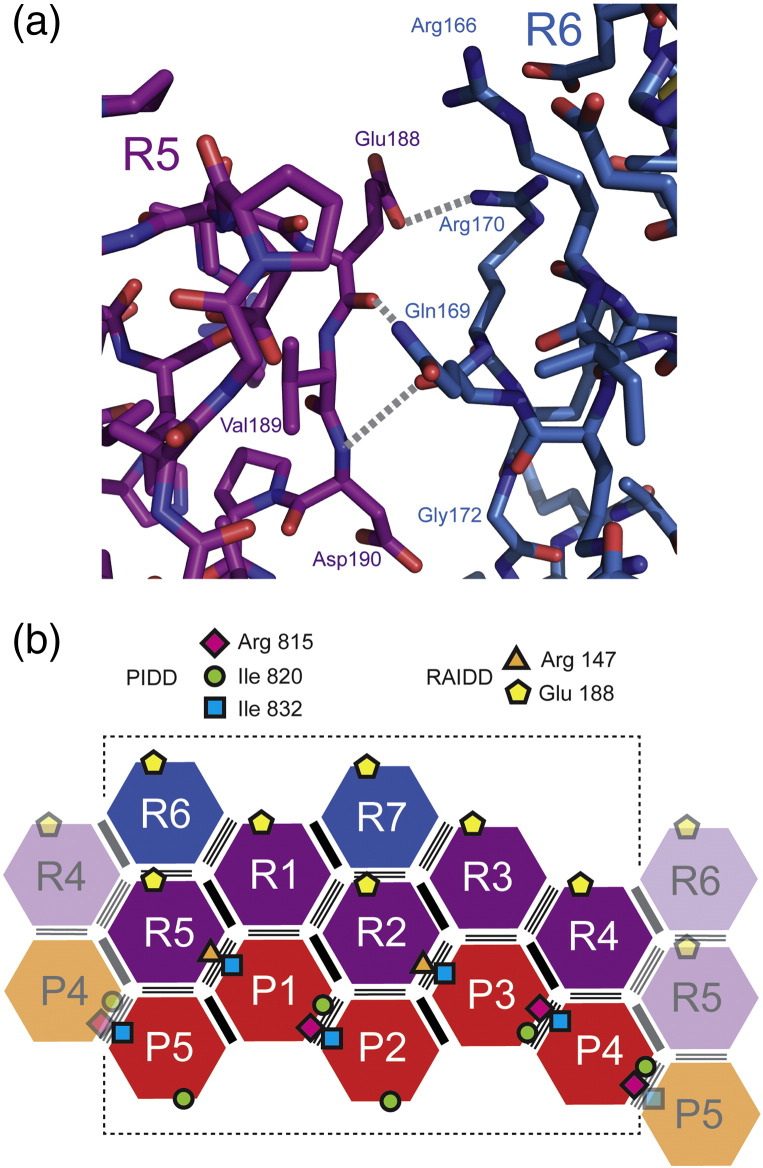
Analysis of interdomain contacts within the PIDDosome core crystal structure. (a) Close-up of the involvement of Glu188 in the type II contact between RAIDD-DD domains R5 and R6. The chains are colored according to the scheme in Fig. 2. Broken lines indicate predicted hydrogen bond interactions. (b) Schematic representation of the position of selected side chains within the PIDDosome crystal structure. Note that Glu188 is only involved in interdomain contacts for RAIDD-DD chains R2 and R5; for R1, R3-R4 and R6-R7, the Glu188 side chain is directed into solvent.

We anticipated that the PIDDosome core complex formed in solution might possess similar characteristics to that visualized in the crystal structure [2] and therefore be intrinsically asymmetric. However, our SEC-MALS and MS data indicated that the sample contained a mixture of species with sizes up to and including the particle with 7:5 RAIDD-DD:PIDD-DD composition that is present in the crystals. We sought to use the crystal structure as a guide to develop a more homogeneous variant of the complex. We noted that the *B*-factors of the two “apical” RAIDD-DD domains in the crystal structure, denoted R6 and R7, are systematically much higher than those for the other 10 chains in the complex (mean *B*-factors for RAIDD-DD domains R1-R7, respectively: 116, 110, 96, 101, 111, 177 and 197 Å^2^; mean *B*-factors for PIDD-DD domains P1-P5, respectively: 101, 116, 94, 92 and 115 Å^2^). We hypothesized that these two domains might be less stably associated with the remainder of the particle and that loss of these two domains might be linked to the observation of ion series corresponding to RAIDD-DD:PIDD-DD compositions of 6:5 and 5:5 in the MS data. Careful analysis of the type II RAIDD:RAIDD interfaces that mediate the attachment of the R6 and R7 RAIDD-DD molecules to the rest of the structure suggested that selective disruption of these contacts might be achievable through targeted site-directed mutagenesis (Fig. 7a). Specifically, we identified that, of the residues that contribute to the type II R:R interface, only Glu188 is not involved in other RAIDD-DD:RAIDD-DD or RAIDD-DD:PIDD-DD contacts (Fig. 7b). For example, the relative solvent-accessible area for the Glu188 side chain in the RAIDD-DD polypeptides R1 through R7 is 99%, 54%, 99%, 88%, 56%, 104% and 103%, respectively. The low values of the accessible surface area for this residue in chains R2 and R5 are consistent with their involvement in type II contacts with chains R6 and R7, respectively. We concluded that variation of the side chain at the Glu188 position had the potential to selectively modify the strength of interaction of R6 and R7 domains in the PIDDosome core. We mutated RAIDD-DD Glu188 to Lys. The RAIDD-DD (E188K) protein was expressed and purified as a stable, folded protein. The ^15^N,^1^H-HSQC spectrum of the RAIDD-DD (E188K) shows very few differences compared to the wild type, consistent with the highly exposed nature of this residue in the free protein (data not shown; see also Fig. 9a and b). We then formed a “mutant” complex of RAIDD-DD (E188K) with PIDD-DD and analyzed the samples as for the wild-type situation.

**Fig. 8:**
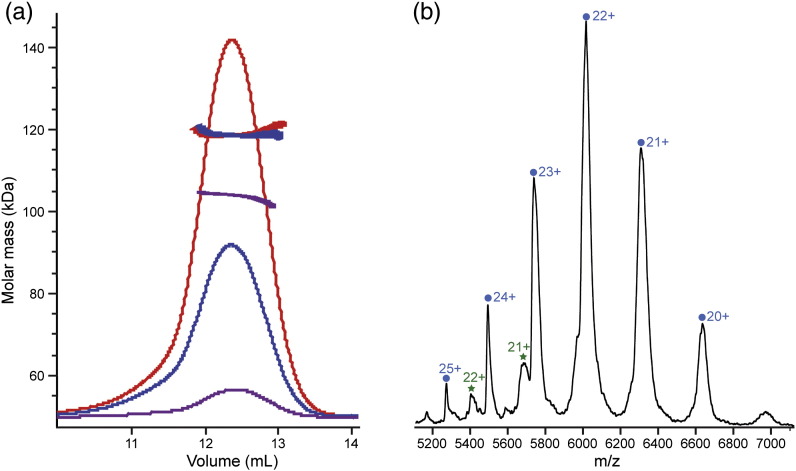
Assessment of the molecular mass of the RAIDD-DD (E188K):PIDD-DD complex in solution by (a) SEC-MALS and (b) nanoESI-MS. In (a), the overall protein loading concentration was 1.5 mg/mL (magenta), 12 mg/mL (blue) and 24 mg/mL (red). In (b), mass spectrum of 15 μM complex in 50 mM ammonium acetate buffer (pH 7.8) and ion series corresponding to the major RAIDD-DD:PIDD-DD species with 5:5 stoichiometry are labeled with charge states, indicated with blue circles. The presence of a low concentration consistent with 5:4 stoichiometry is indicated (green stars).

We assessed the molecular mass of the mutant complex by both SEC-MALS and nanoESI-MS. SEC-MALS samples were run over a total protein concentration range of 1.5–24 mg/mL. The symmetric chromatographic light-scattering profile was very similar to the one obtained for the PIDDosome core complex (Fig. 8a). The analysis of the SEC-MALS data over a concentration range of 12–24 mg/mL yielded an average molecular mass of ~ 120 kDa corresponding to nine chains similar to the previous results obtained from wild-type PIDDosome core complex. However, the minimum average molecular mass at 1.5 mg/mL concentration was ~ 104 kDa corresponding to eight chains and slightly higher than that observed for the wild-type PIDDosome core complex.

The purified mutant complex was also subjected to nanoESI-MS analysis. The mass spectrum again showed signals corresponding to high *m*/*z* ion series attributable to the PIDDosome particles. There were two overlapping charge series of ions. These series represent assemblies with overall masses of 119 kDa and 132 kDa corresponding to 5:4 and 5:5 RAIDD-DD:PIDD-DD stoichiometric ratios based on the internal mass references for the isolated monomers (Fig. 8b). The population distribution for the two complexes was 13% and 87%, respectively, and the recovered molecular masses were within 3% of the predicted values based on the amino acid sequence.

Taken together, the intact MS data suggest that the RAIDD-DD (E188K) mutation alters the composition of the particles formed in combination with PIDD-DD to yield a dominant species with 5:5 stoichiometric ratio, consistent with the notion that two RAIDD-DD domains are relatively loosely bound into the wild-type particle.

### Cross-peak splitting and cluster formation is an indication of asymmetric properties of the complex

**Fig. 9:**
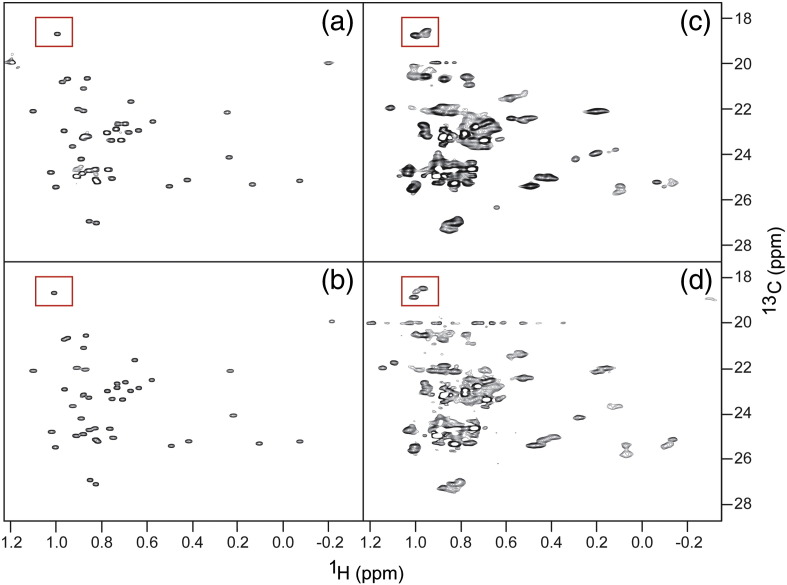
Comparison of the ^13^C-methyl-TROSY NMR spectra of wild type (a and c) and RAIDD-DD (E188K) (b and d) in the absence and presence of excess PIDD-DD. The positions of the cross-peaks of unbound RAIDD-DD (E188K) are essentially identical with those of the wild type. However, notable differences in the cross-peak clusters corresponding to the well-resolved RAIDD-DD Val189-C^γ2^H_3_ cross-peak (highlighted by red rectangles) are detected, presumably reflecting both the proximity to the mutation site at residue 188 and the different overall stoichiometry of the complex formed.

We separately examined the complex formed between RAIDD-DD (E188K) and PIDD-DD using ^15^N-HSQC and ^13^C-methyl-TROSY NMR in the same way as for the wild type. The results of the ^15^N-HSQC experiments of the ^15^N-labeled RAIDD-DD (E188K) were almost identical with those of the wild-type RAIDD-DD, consistent with formation of a high-molecular-weight species for which the transverse nuclear relaxation rates lead to loss of signal for all but the most mobile regions of the component protein domains (data not shown). The corresponding ^13^C-methyl-TROSY experiments results are presented in Fig. 9: (a) and (b) depict the ^13^C-methyl-TROSY spectra of isolated wild type and mutant ILV-RAIDD-DD, respectively, and (c) and (d) show the spectra of the complexes formed in each case in the presence of excess PIDD-DD. The principal observation is that, as for the wild-type case, the behavior of the cross-peaks is divisible into two classes: a subset remains relatively unperturbed in chemical shifts and broaden only slightly and a second subset is transformed into clusters of cross-peaks with resolved or partially resolved chemical shifts and much lower intensity. Overall, the pattern of behavior and the nature of the chemical shift perturbations for the second subset of cross-peaks are highly similar to those observed for the wild-type complex. Figure 9 illustrates that, in spectra of the wild-type and mutant ILV-RAIDD-DD:PIDD-DD complexes, the effects of complexation are highly similar. However, careful inspection shows that, despite the high degree of overlap in the spectrum, it is possible to identify subtle differences between the cross-peak clusters in the spectra. An example of these differences is provided by the RAIDD-DD Val189-C^γ2^H_3_ cross-peak highlighted by the red boxes in Fig. 9. Val189 residue neighbors the mutation site at position 188, within the α5–α6 loop with the side chain partially buried in between the flank of the α5 helix and the N-terminal end of the α6 helix (Fig. 7a). The relative solvent-accessible surface area for this residue in RAIDD-DD chains R1 through R7 is 22%, 19%, 24%, 27%, 7%, 13% and 18%, respectively. The chemical shifts of the Val189-C^γ2^H_3_ cross-peak are essentially insensitive to the Glu188Lys mutation in the isolated RAIDD-DD protein—the centers of the two cross-peaks differ by less than the line widths. However, on formation of the complex with PIDD-DD, the pattern of the local cluster of derivative cross-peaks, close to the chemical shifts of the original Val189 signal, is noticeably different between the case of wild-type and mutant RAIDD-DD:PIDD-DD complexes (cf. peak clusters highlighted in Fig. 9c and d).

## Discussion

We have described the analysis of the molecular weight and subunit stoichiometry of the PIDDosome core particles using SEC-MALS, intact MS and heteronuclear NMR spectroscopy. Consistent with expectations from previous work, we found that the combination of RAIDD-DD and PIDD-DD in solution leads to the formation of multimeric complexes with molecular masses above 100 kDa. Our observations can be described in terms of the formation of R*_m_*:P*_n_* particles in which R and P correspond to the DDs of RAIDD and PIDD, respectively, and *m* and *n* are integers corresponding to the numbers of each domain in the complex. At relatively high protein concentration, the SEC-MALS results for both wild-type and mutant R*_m_*:P*_n_* particles using SEC-MALS (Figs. 3a and 8a) suggest a composition comprising a total of nine chains for both wild-type and mutant PIDDosome core complexes. The staining pattern obtained from SDS-PAGE separation of the complexes indicated an approximately equal amount of PIDD-DD and RAIDD-DD molecules in the complex. Coupled with knowledge of the individual domain masses that differ by only 0.4 kDa (~ 3.1%), we would predict quasi-equimolar composition for the particles. On their own, these results would lead us to conclude that the particles detected using SEC-MALS analysis corresponds to the formation of either R_4_:P_5_ or R_5_:P_4_ particles. At lower protein concentration (1.5 mg/mL), the predicted masses for the eluting complexes are somewhat lower: 93 kDa for the wild type and 104 kDa for the mutant corresponding to compositions of R_4_:P_3_, R_3_:P_4_ or R_4_:P_4_, respectively.

When the PIDDosome core complexes were characterized using MS, the data similarly indicated the potential for flexible stoichiometry. Namely, the data for the wild-type complex indicate the presence of species corresponding to R_6_:P_5_ (43%), R_7_:P_5_ (29%) and R_5_:P_5_ (28%) (Fig. 3b). For the mutant complex, the particles were significantly lighter, and the sample had a lower degree of compositional dispersion: R_5_:P_4_ (13%) and R_5_:P_5_ (87%) (Fig. 8b). Intriguingly, the masses measured do not correspond precisely to that expected for the R_7_:P_5_ complex detected both *in crystallo* and visualized by electron microscopy [2].

Taken together, the SEC-MALS and nanoESI-MS clearly indicate that the PIDDosome core particles can possess a flexible composition in solution and that perhaps the particles contain a “stable” core of seven, eight or as many as ten domains that can be appended at higher protein concentrations by additional PIDD-DD and, more likely, RAIDD-DD domains. In this picture, the R_7_:P_5_ complex that can form into a crystal lattice corresponds to only the heaviest of a series of different particle types that can be detected in solution.

The apparent discordance between the SEC-MALS and the MS measurements presumably reflects an aspect of the behavior of this type of system. It has been reported before that the mass of a binary DD complex appears to be lower by SEC-MALS than by nanoESI-MS [33]. A confounding aspect of the SEC-MALS measurements might be that the concentration of the sample effectively diminishes by diffusional “spreading” as it moves down the column matrix, making the measurement technically a non-equilibrium one. Thus, the instantaneous concentration of the analyte in the eluant at the detector is significantly lower than that which was loaded at the start. For complexes that form with a high degree of cooperativity or small equilibrium dissociation constant, such dilution may not greatly affect the molecular mass measurement. For complexes demonstrating either weak cooperativity or high dissociation constant, then it is possible that the SEC-MALS methodology may lead to underestimation of the equilibrium molecular mass.

We adopted specific isotope labeling and heteronuclear NMR spectroscopy to probe the nature of the PIDDosome core complexes. Both ^1^H,^15^N-HSQC and methyl-TROSY experiments reveal that signals from the flexible terminal regions of the proteins remain relatively sharp with no significant chemical shift perturbations. For the corresponding resonances, the high molecular mass and slow overall tumbling of the particles does not significantly contribute to the line width. These resonances are in the fast exchange regime between the essentially identical chemical shifts for free and complex states [33]. On the other hand, the cross-peaks from the globular regions are either broadened to the noise level in the case of the ^15^N-HSQC measurements or split into clusters of cross-peaks with evidence for chemical shift heterogeneity and diminished peak intensities in the case of the ^13^C-methyl-TROSY experiments. The precise nature of the exchange regime that is operative in the ^15^N-HSQC experiments is not clear since we have been unable to detect the bound-state signals and thereby identify the chemical shift differences (Fig. 4). Stepwise addition of PIDD-DD to ^15^N-RAIDD-DD or RAIDD-DD to ^15^N-PIDD-DD leads to systematic diminution of the intensity of the free-state cross-peaks in the ^15^N-HSQC spectrum, suggesting that the system displays slow exchange between the narrow signals representative of the ^15^N-labeled monomer, as well as invisible broad signals of the bound complex. Attempts to recover the signals of the bound state using ^15^N,^2^H-labeled proteins and ^15^N,^1^H-TROSY methods did not yield any further information to resolve the basis for the appearance of the ^15^N-HSQC spectra of the complexes.

ILV-labeling strategies [34] and minimization of transverse relaxation effects for methyl groups using ^13^C-methyl-TROSY NMR [24,39,40] make it possible to record high-quality solution NMR spectra of high-molecular-mass complexes [24,25]. Typically, application of ^13^C-methyl-TROSY to appropriately isotope-labeled proteins yields spectra that exhibit good sensitivity and resolution, even in instances where the corresponding ^15^N,^1^H-NMR spectra are intractable. When applied to the PIDDosome core complex, the resulting spectra are distinctly more complex that would be expected for a multimeric system with symmetric quaternary structure. Not unexpectedly, the methyl groups of Ile, Leu and Val residues in the N- and C-termini flexible regions yield essentially unperturbed “singlet” cross-peaks consistent with maintenance of a high degree of disorder in the complex (Fig. 5). On the other hand, the spectra clearly suggest that individual methyl groups within the globular regions of both RAIDD-DD and PIDD-DD occupy multiple distinct environments leading to a multiplicity of ^1^H and ^13^C chemical shifts and clusters of resolved or partially resolved cross-peaks in the 2D NMR spectrum. In principle, the origin of such complexity could arise either from the presence of asymmetry in the complex or from compositional dispersion of the particles in solution.

Having obtained evidence for the compositional dispersion by SEC-MALS and MS measurements of the PIDDosome core complex, we sought to minimize this property by consideration of the crystal structure. Reflecting on the pattern of *B*-factors in the crystal structure that suggested some flexibility or disorder in the R6 and R7 RAIDD-DD domains, coupled with the relatively select nature of the type II contacts between domains R5 and R6, as well as domains R2 and R7, we probed the effect of mutating the residue Glu188 in RAIDD-DD. Glu188 in R5 and R2 makes hydrogen bond and electrostatic contact with both Gln169 and Arg170 in R6 and R7, whereas in R1, R3-R4 and R6-R7, Glu188 is highly exposed to solvent. Therefore, mutation of this residue was expected to impact only the R5:R6 and R2:R7 interfaces and leave unchanged all other types I–III R:R and R:P contacts. Mutation of Glu188 to Lys clearly lowered both the overall molecular mass and compositional dispersion of the PIDDosome particles formed, as assessed by nanoESI-MS. By far, the dominant species in solution under these conditions is the R_5_:P_5_ heterodecamer.

Despite the effects of the RAIDD-DD (E188K) mutation on the apparent mass of the particle, the ^13^C-methyl-TROSY NMR spectrum of the mutant PIDDosome complex is, for the most part, indistinguishable from that of the wild-type complex (Fig. 9). The characteristic cross-peak clusters are still present. The fact that the overall character of the spectrum is barely changed argues that its complexity derives from low or absent symmetry of the complex, as opposed to compositional dispersion. Moreover, the fact that differences between the spectra are restricted to cross-peaks whose residues are close to the mutation site (e.g., Val189; Fig. 9) is consistent with successful disruption of the contacts involving the “apical” domains R6 and R7 and that the spectrum of the mutant complex is that of the R_5_:P_5_ “core” We note that, while it is strictly impossible to conceive of a structure with composition R_7_:P_5_ that possesses overall symmetry, in principle, a particle with composition R_5_:P_5_ could possess cylindrical symmetry (an R_5_ ring sitting on a P_5_ ring) that would yield degenerate chemical shifts for any given nucleus. The fact that the spectrum of the mutant PIDDosome core particle displays non-degenerate chemical shifts argues that the structure of the particle in solution is closely similar to that of the corresponding R_5_:P_5_ portion of the wild-type crystal structure. Close inspection of the spectrum indicates that each cross-peak cluster comprises at least four components and the shape of the individual components sometime hints at a close overlap of two (or more) (Fig. 7). Thus, it is conceivable that each cluster comprises at most five components consistent with the five unique environments predicted for a R_5_:P_5_ particle that lacks symmetry. For example, in Fig. 6b, it appears that, in the complexed state, the δ1-methyl-group cross-peak of PIDD-DD Ile820 forms a cluster of three features at minimum (with ~ 2:1:2 peak intensity ratio) and the corresponding cross-peak of PIDD-DD Ile832 forms a cluster of four features (with ~ 2:1:1:1 intensity ratio). In the crystal structure, the Ile820 residue is on the solvent-exposed surface of domains P2 and P5, and for P1, P3 and P4, it is involved in type III interactions with P2, P4 and P5, respectively (schematically represented in Fig. 7b). Ile832 is involved in type III interactions for all of P1-P5, in three cases being juxtaposed with Arg815 in the neighboring PIDD-DD domain (P2:P5, P4:P3 and P5:P4 contacts) and in the other two cases being juxtaposed with Arg147 from a neighboring RAIDD-DD domain (P1:R5 and P3:R2 contacts) (Fig. 7b). Given the level of asymmetry, it would be not unexpected that the cross-peaks corresponding to individual sites in the structure could display different chemical shifts. The fact that the cross-peak clusters comprise multiple subpeaks suggests that any exchange of protein chains between sites with the particle is slow on the chemical shift timescale.

We note that the line widths and peak intensities exhibited within a given cluster and, indeed, across clusters for a given residue type appear non-uniform. Notwithstanding the potential for accidental overlap of cluster components, differential line broadening of signals in ^13^C-methyl-TROSY spectra might also arise due to the precise nature of dipolar interactions between the protons of a given methyl group with the “external” protons from other methyl groups (assuming no other proton to be present in the protein chain or bulk solvent) [41]. In an asymmetric structure, since the environments of a given residue in the, say, RAIDD-DD chains, are different (albeit sometimes subtly) within the overall particle structure, the potential exists for differential contributions of ^1^H–^1^H interactions to the resonance line widths.

Instances of heterogeneity in the NMR spectra of purified proteins have been reported before but within rather different contexts to that reported here. “Trivial” examples of chemical shift non-degeneracy can occur in the event of conformational exchange processes such as *cis*–*trans* isomerization of Xxx-Pro peptide bonds or inversion of the stereochemistry within disulfide bonds [42,43]. Homodimeric proteins are almost universally observed to yield a single set of cross-peaks in the NMR spectrum, consistent with either the formation of a symmetric homodimer in solution or to the fast exchange of two asymmetric conformers within a AB ⇌ BA equilibrium. An example of the latter case has been reported for the leucine zipper domain of the DNA-binding protein c-Jun where a pair of asparagine residues switches between two self-similar hydrogen bonding arrangements [44]. A more extreme case of a AB ⇌ BA equilibrium is presented by the integral membrane transporter EmrE from *Escherichia coli* prepared in lipid bicelles [45]. In this example, the exchange is slow on the NMR timescale, and as a result, each NMR active nucleus in the protein exhibits two sets of chemical shifts, yielding doubled cross-peaks in multidimensional NMR data. Evidence for the exchange between the two structurally identical structures in the asymmetric dimer is provided in the form of ZZ-exchange correlations and the pattern of paramagnetic relaxation enhancement effects induced in the presence of a water-soluble lanthanide reagent. More common instances of chemical shift heterogeneity arise due to the effect of symmetry breaking when a symmetric homomer protein binds to a single target molecule, such as in the case when the RNA-binding domain of the bacterial protein LicT (also known as co-antiterminator, CAT) forms a complex with the ribonucleic antiterminator (RAT) target [46]. In isolation, the CAT protein forms a symmetric homodimer with a single set of NMR cross-peaks, whereas when bound to the RNA partner in a 2(CAT):1(RAT) complex, the number of features in the protein NMR spectrum was doubled, reflecting its intrinsic asymmetry. Analogous behavior was observed for the complex of the homodimeric *Bacillus subtilis* replication terminator protein with a 21-base-pair, double-stranded DNA target [47]. Similarly, it has been reported that addition of a protein ligands to the homodimeric heat shock 90 protein leads to peak doubling attributed to symmetry breaking, evidenced in the methyl-TROSY spectra [48,49].

AB ⇌ BA equilibria and symmetry breaking do not appear to be relevant to the PIDDosome core NMR data. Rather, it would be necessary to appeal to homomeric systems that display non-equivalent environments in the separate protomers, such as *n*-mers (*n* greater than 3) that exhibit parallel helical symmetry. For example, NMR studies of peptides designed to mimic collagen yield non-degenerate chemical shifts for equivalent atoms in the leading, middle and trailing strands [50,51]. To our knowledge, no instance of an application of ^13^C-methyl-TROSY NMR has yet been reported for a system with helical symmetry. Rather, the closest that such applications have come to an asymmetric system is the observation that, in the homoheptameric 20S core particle, proteasome methionine residues in the N-terminal region of the α subunits can exchange between locations inside and outside of the catalytic chamber, characterized by different chemical shifts in the methyl-TROSY spectrum [52]. On average, only two of the flexible termini are in the “in” state at any one time, and thus, the structure is formally non-symmetric.

Detailed examination on the crystal structure of the PIDDosome core reveals the presence of pseudohelical symmetry [53]. Thus, the authors of the work describe that the pattern of P-R heterodimeric “subcomplexes” is arranged in five successive screw rotations around a central (vertical) axis; the screw rotations are of two types, one of 84° and translating down the axis (three occurrences) and the other one of 54° translating up the axis (two occurrences) (Fig. 2b). Alternatively, the structure can be described as a double-stranded left-handed helical oligomer, where each of the strands contains both PIDD-DD and RAIDD-DD subunits [53]. Despite these elements of pseudosymmetry, one would still predict from this structure the potential for non-degenerate chemical shifts for the different PIDD-DDs and RAIDD-DDs particularly for residues close to any of the domain–domain interfaces. Thus, it seems reasonable to presume that the experimentally observed ^13^C-methyl-TROSY spectrum reflects the formation of a structure in solution that is either identical with or very closely similar to that observed in the crystal.

The overall pattern of behavior in the NMR spectra for the PIDDosome core complexes is broadly similar to that observed for the complex formed between DDs of CD95 and FADD thought to correspond to the core of the DISC (*d*eath-*i*nducing *s*ignaling *c*omplex) [33]. In the latter case, the data were less clear-cut since the spectra always contained residual intensity for the labeled “free” domain, even in the presence of excess binding partner. Thus, the formation of the PIDDosome core complex would appear to have a higher affinity and a higher degree of cooperativity. Nevertheless, the correspondence of the NMR spectra and crystal structure of the PIDDosome core reported here supports the interpretation that the NMR spectra of the DISC core particle are well explained by a model based on the crystal structure of the PIDDosome [33,37] rather than an alternative structure with discordant molecular mass and other characteristics [54] or on the basis of compositional dispersion. The NMR spectra of both of these complexes provide the basis for further investigations into the mechanism and kinetic aspects of particle assembly using a combination of NMR and intact MS methods.

## Experimental Procedures

### Plasmid construction and mutagenesis

We use the human PIDD-DD (778–883) and RAIDD-DD (99–199) corresponding to the DDs, consistent with those used for the crystal structure 2OF5 [2]. DNA encoding the DD of PIDD and RAIDD was separately inserted into a pET26b vector that encodes a non-cleavable C-terminal His_6_ affinity tag residues. Point mutant RAIDD-DD construct was created using QuikChange site-directed mutagenesis.

### Protein expression, purification and complex formation

The PIDD-DDs and RAIDD-DDs were expressed in *E. coli* BL21(DE3) cells at 20 °C and purified using metal affinity purification (Ni-IDA) followed by SEC with final elution into 20 mM Tris–HCl (pH 8.0), 50 mM NaCl, 3 mM NaN_3_ and 0.5 mM tris(2-carboxyethyl)phosphine (TCEP) hydrochloride. The samples were mixed and incubated at room temperature for 1 h before subjecting them to SEC using Superdex S200 HR 10/30 (GE Healthcare). The complex was eluted at 12.5 mL and was concentrated to 24 mg/mL.

Uniform ^15^N isotope, ^13^C labeling and ^2^H labeling were achieved using standard protocols in M9 media. Uniform ^2^H-Ile-δ_1_(^13^CH_3_), Leu-δ, Val-γ(^13^CH_3_) labeling of PIDD and RAIDD proteins for ILV ^13^CH_3_-TROSY NMR experiments was performed in 99% D_2_O M9 medium containing (^2^H,^12^C)-glucose as the sole carbon source. To introduce methyl labels, we supplemented the medium with 60 mg/L of 2-keto-3,3-*d*_2_-4-^13^C-butyrate acid and 100 mg/L of 2-keto-3-methyl-*d*_3_-3-*d*_1_-4-^13^C-butyrate for Ile and Leu/Val, respectively, 1 h before induction. Proteins for ^13^CH_3_-TROSY NMR experiments were exchanged into NMR buffer [20 mM Tris–HCl (pH 8.0), 50 mM NaCl and 0.5 mM TCEP HCl] in D_2_O.

### Multi-angle light scattering

Analytical SEC-coupled MALS was recorded at 16 angles using a DAWN-HELEOS laser photometer (Wyatt Technology) and differential refractometer (Optilab rEX, Wyatt Technology). Flow cell was maintained at 25 °C (Wyatt Technology). Complexes were isolated by semi-preparative S200 size-exclusion column and concentrated in buffer A. Samples (100 μL) were injected onto a Superdex 200 HR 10/30 size-exclusion column (GE Healthcare) equilibrated buffer containing 20 mM Tris–HCl (pH 8.0), 50 mM NaCl, 3 mM NaN3 and 0.5 mM TCEP. The flow rate was 0.5 mL/min. Data were collected and analyzed using the program ASTRA 5.1 as previously described (Ivins *et al*., 2009).

### Intact MS

NanoESI-MS measurements were performed in the positive ion mode on a modified Q-ToF II mass spectrometer (Waters) [55], using in-house prepared, gold-coated glass capillaries [47]. The MS parameters in order to preserve non-covalent interactions were as follows: capillary voltage, 1.5 kV; cone voltage, 80 V; extractor, 5 V; source backing pressure, 6–8 mbar; collision cell pressure, 5 psi. Collision cell energy was between 60 and 100 V. Measurements were taken for intact protein complexes prepared for SEC-MALS analysis and exchanged into 50 mM ammonium acetate (pH 7.8) using Biospin 6 microspin columns (Bio-Rad Laboratories). Data were processed using our in-house software Massign [50]. The results are representative of multiple independent experiments.

### NMR spectroscopy

All spectra were recorded at 25 °C on Varian INOVA or Bruker AVANCE spectrometers operating at 14.1 T, 16.5 T and 18.8 T. Data were processed with NMRPipe/NMRDRAW (Delaglio, 1995) and analyzed with CCPN software (Vranken *et al*., 2005). Resonance assignments for PIDD and RAIDD proteins were obtained using standard protocols. Titrations of a labeled protein (0.2 mM) with its unlabeled partner (0–1 molar equivalents) were conducted at constant concentration of the labeled component (McAlister *et al*., 1996). Typical acquisition time for 2D WATERGATE-flipback ^15^N,^1^H-HSQC (Grzesiek and Bax, 1993) and ^13^CH_3_-TROSY [39] data sets was about 1 h and 3 h, respectively. Relaxation delay for ^13^CH_3_-TROSY experiments was 1.3 s.

### Solvent-accessible surface calculations

Relative side-chain solvent-accessible surface calculations were performed using NACCESS (Hubbard SJ and Thornton JM, 1993), Computer Program, Department of Biochemistry and Molecular Biology, University College London).
